# IMP3 is a biomarker for non-muscle-invasive urothelial carcinoma of the bladder associated with an aggressive phenotype

**DOI:** 10.1097/MD.0000000000016009

**Published:** 2019-07-05

**Authors:** Feiya Yang, Qiang Zhou, Lingquan Meng, Nianzeng Xing

**Affiliations:** a Department of Urology, National Cancer Center/ National Clinical Research Center for Cancer/ Cancer Hospital, Chinese Academy of Medical Sciences and Peking Union Medical College, Beijing; b Department of Urology, Zhongnan Hospital of Wuhan University, Wuhan; c Department of Urology, Beijing Chaoyang Hospital, Capital Medical University, Beijing; d Department of Urology, Qianfoshan Hospital, Shandong University, Jinan, PR China.

**Keywords:** biomarker, IMP3, non-muscle-invasive urothelial carcinoma of bladder, prognosis, survival

## Abstract

Bladder cancer is one of the most common malignancies of urinary tract. The current study aimed to investigate the role of insulin-like growth factor II messenger RNA binding protein 3 (IMP3) expression in the prognostic evaluation of non-muscle- invasive urothelial carcinoma of the bladder.

Immunohistochemistry (IHC) was carried out to examine IMP3 protein expression in specimens from 183 cases of non-muscle-invasive urothelial carcinoma, 20 cases of muscle-invasive urothelial carcinoma and 20 benign tissues adjacent to cancer tissue.

The expression of IMP3 was not detected in the adjacent benign tissues. The expression intensity of IMP3 in muscle-invasive samples was significantly higher than that in non-muscle-invasive urothelial carcinoma specimens (*P* = .008). IMP3 expression was significantly related with advanced tumor stage (*P* < .001), advanced tumor grade (*P* = .004), and tumor recurrence (*P* < .001) in non-muscle-invasive urothelial carcinomas. Kaplan–Meier analysis showed that IMP3-positive patients had much lower disease-free (*P* < .001), progression-free (*P* = .002) and metastasis-free (*P* = .019) survival rates compared with those with IMP3-negative tumors. By multivariable Cox analysis, we also found that IMP3 expression in non-muscle- invasive urothelial carcinomas proved to be an independent unfavorable prognostic factor of disease-free survival (HR: 2.52; 95% CI, 1.39–4.56; *P* = .002), progression- free survival (HR: 5.19; 95% CI, 1.54–17.46; *P* = .008) and metastasis-free survival (HR: 4.87; 95% CI, 1.08–22.02; *P* = .040).

Our results demonstrate that the expression of IMP3 in non-muscle- invasive bladder cancer can serve as an independent predictor that will help recognize the subgroup of patients with a high ability to relapse, progress, and metastasize and who might get the maximum benefit from an early and more aggressive treatment strategy.

## 1 Introduction

Bladder cancer is one of the most common malignancies of the urinary tract, with an estimated 81,190 new cases per year in the United States,^[1]^ of which over 95% are urothelial carcinomas.^[2]^ Of all patients diagnosed with bladder cancer, up to 70% have non-muscle-invasive tumors at initial presentation. Transurethral resection and intravesical chemotherapy are the preferred initial management for non-muscle-invasive tumors. However, approximately 70% of these patients will relapse, and as many as 10% to 30% will develop to invasive disease after treatment.^[3]^ This indicates that patients with non-muscle-invasive tumors represent a heterogeneous group regarding their prognosis. Predicting the prognosis of bladder cancer accurately, so as to provide further suitable treatment for patients with great potential risk of progression, is particularly important. The variability of clinical outcomes in patients with identical morphologies probably reflects differences in molecular backgrounds, which leads to research of molecular markers for detecting tumor biological characteristics and guiding clinical diagnosis and treatment. This has become a very interesting area.

IMP3 is a member of the insulin-like growth factor II messenger RNA binding protein (IMP) family, which includes IMP1, IMP2, and IMP3.^[4]^ In the early processes of embryogenesis, IMP family members can bind target mRNAs with high specificity and play a significant role in RNA transportation and stability and cell proliferation and migration.^[5]^ The IMP3 gene is located at chromosome 7p11.2 ± 11cM,^[6]^ the same position as KOC (k-homology region controlling protein overexpressed in malignant tumors), discovered in pancreatic cancer tissues in 1996.^[7]^ Many studies have suggested that IMP3 is highly expressed in a variety of malignant tumors but not in adjacent benign tissues.^[8–14]^ Moreover, in vitro studies have confirmed that IMP3 plays a decisive role in promoting proliferation and invasion of human K562 leukemia and Hela cells,^[15,16]^ indicating that IMP3 may be involved in the development of malignant tumors.

In the current study, we detected the expression of IMP3 in non-muscle-invasive urothelial carcinoma of the bladder, explored its correlation with tumor clinical characteristics, and evaluated its role in diagnosis and prognosis.

## 2 Materials and methods

### 2.1 Specimens and clinical characteristics

Formalin-fixed, paraffin-embedded specimens were obtained from 183 patients with non-muscle-invasive bladder urothelial carcinoma who underwent transurethral resection for the first time at our hospital from October 2010 to September 2013. The archives of specimens and clinical follow-up information for all patients were readily available. We also excluded patients who had a history of other tumors and carcinoma in situ and received chemotherapy before surgery. The 183 non-muscle-invasive bladder urothelial carcinoma patients included 144 males and 39 females, and the mean age was 65.6 years (range from 28 to 84). The pathological results indicated that 137 cases were stage Ta and 46 cases were stage T1. In addition, 32 cases were papillary urothelial neoplasm of low malignant potential (PUNLMP), 88 cases were low grade, and 63 cases were high grade. The mean tumor size was 2.4 cm. Of all patients, 143 had a single tumor and 40 had multiple tumors. We analyzed 20 muscle-invasive samples and 20 benign tissues adjacent to cancer for a direct comparison of IMP3 expression. Of the 20 muscle-invasive carcinoma patients, 14 were male, and 6 were female; the mean age was 66.0 years (from 50 to 80 years). Of these cases, 17 were stage T2, 1 was stage T3, and 2 were stage T4. Of the 20 patients with benign tissues adjacent to cancer, 15 were male, and 5 were female; the mean age was 65.0 years (from 48 to 79 years). Tumor grade and stage were reassigned according to the 2004 World Health Organization grading system and the 2009 TNM staging system, respectively. This study was approved by the Research Ethics Committee of Beijing Chaoyang Hospital (China).

### 2.2 Immunohistochemistry (IHC) analysis

IHC staining for IMP3 was performed as previously described.^[17]^ Briefly, formalin-fixed, paraffin-embedded tissue blocks were cut into 5-μm sections and mounted on positively charged slides. Xylene was used to deparaffinize the tissue sections followed by rehydration with graded alcohol solutions. Then, sections were incubated in citrate buffer (pH 6.0) at 210°C for 10 minutes and at 160°C for 5 minutes in a pressure cooker. After incubation in 3.0% hydrogen peroxide for 10 minutes and washing in PBS, the tissue sections were immersed in a working solution of anti-IMP3 antibody (prediluted, ab82044, Abcam, 1:5 dilution) for 1 hour at 37°C. Tissue sections were exposed to a secondary antibody (MaxVisionTM HRP-Polymer anti-rabbit IHC kit, Maixin Bio, Fuzhou, China) for 20 minutes at room temperature. Lastly, the sections were incubated with DAB chromogen and then counterstained with hematoxylin. Positive and negative controls were used throughout all immunostaining protocols.

**Figure 1:**
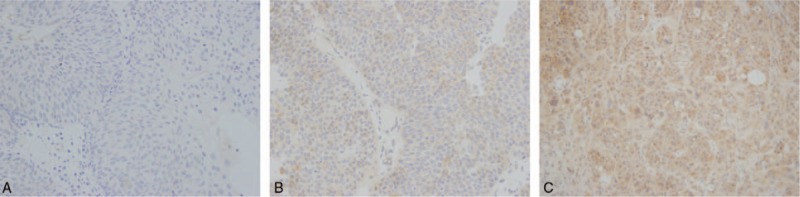
IHC staining of non-muscle-invasive urothelial carcinoma for IMP3. A, negative; B, weak; C, strong.

Tumor cells with dark brown cytoplasmic staining were defined as positive. IHC evaluation was done by 2 physicians who were blinded to the clinical outcome. In the case of IMP3 staining, intensity was scored as 0, 1, 2, or 3, corresponding to achromaticity, light yellow, pale brown, and sepia. In addition, the percentage score was defined as follows: 0% to 5%, 0 points; 6% to 25%, 1 point; 26% to 50%, 2 points; 50% to 75%, 3 points; and 76% to 100%, 4 points. A final histochemical score was calculated by multiplying the intensity score by the percentage score. The ultimate staining scores were categorized as negative (0), weak (<6) and strong (≥6) (Fig. 1).

### 2.3 Statistical analysis

Statistical analysis was performed with SPSS software version 23.0. The associations between IMP3 expression and clinical parameters of the bladder cancer patients were evaluated by using the *χ*^2^ test and Fisher exact test. Independent 2-tailed Student *t* test was also applied to determine the statistical significance of differences in IMP3 expression levels between cancer tissues and benign tissues adjacent to cancer. Survival rates were estimated using the Kaplan–Meier method, and the difference in survival curves was analyzed with the log-rank test. The Cox proportional hazards regression model was applied for multiple analyses. A two-sided test and *P* values of <0.05 were considered statistically significant.

## 3 Results

### 3.1 IMP3 expression in urothelial carcinomas and benign tissues adjacent to cancer

IMP3 expression was not detected in the benign tissues adjacent to cancer. The IMP3 expression detection rate in 183 primary non-muscle-invasive urothelial carcinoma patients was 59.02% (108 of 183), of which 31.15% (57 of 183) had weak expression and 27.87% (51 of 183) had strong expression. In contrast, the IMP3 expression detection rate was 80.00% (16 of 20) in muscle-invasive specimens, of which 20% (4 of 20) had weak expression and 60% (12 of 20) had strong expression (*P* = .008).

### 3.2 Correlation between IMP3 expression and clinical characteristics

As shown in Table 1, the expression of IMP3 was highly related to advanced tumor stage (*P* < 0.001), advanced tumor grade (*P* = .004), and tumor recurrence (*P* < .001) in non-muscle-invasive urothelial carcinomas of the bladder. In contrast, little relationship was seen between IMP3 expression and patient age (*P* = .476), gender (*P* = .508), tumor size (*P* = .326), tumor multiplicity (*P* = .810), and smoking history (*P* = .556).

**Table 1:**
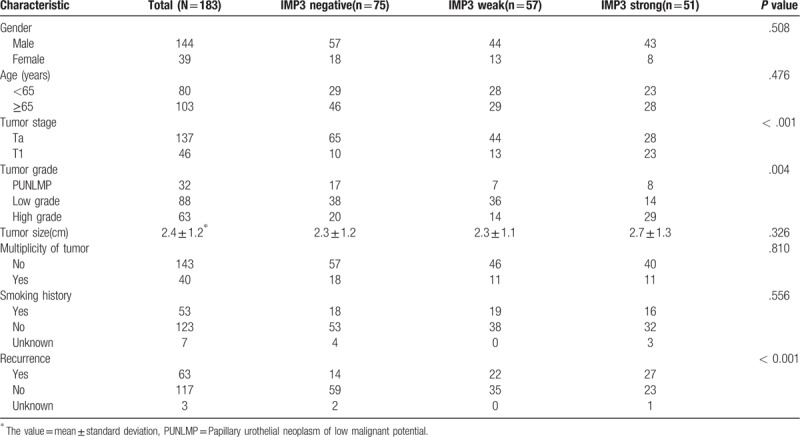
Clinical and pathology characteristic of patients with non-muscle-invasive urothelial carcinoma.

### 3.3 Correlation between IMP3 expression and tumor prognosis

**Figure 2:**
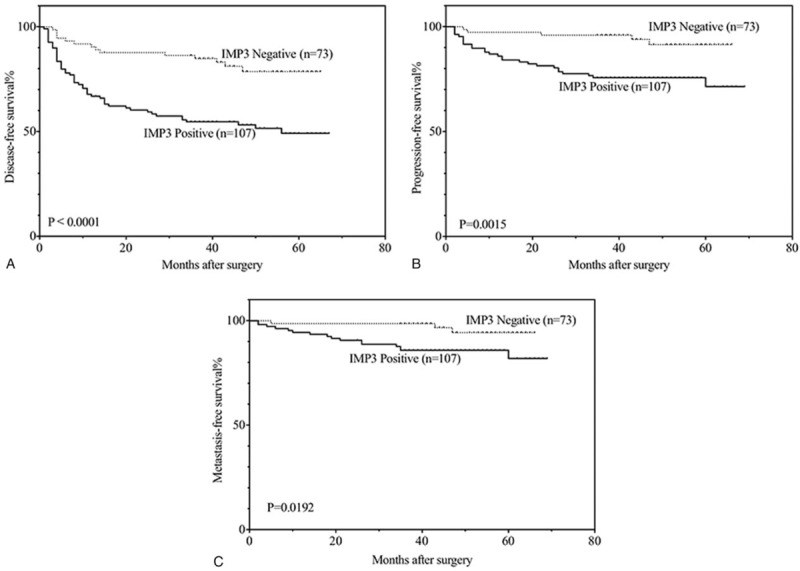
Kaplan–Meier analysis of disease-free (A), progression-free (B) and metastasis-free (C) survival in all patients with non-muscle-invasive urothelial carcinomas.

To further elucidate the role of IMP3 expression in non-muscle-invasive urothelial carcinomas, the overall, disease-free, progression-free and metastasis-free survival rates of 180 patients (3 patients were lost) were analyzed with the Kaplan–Meier method. All patients were followed until August 2016, and the median follow-up was 48 months, ranging from 5 to 69 months. During the follow-up time, 16 of the 180 patients (8.9%) died, of which 4 (4 of 16) died from other causes. A total of 63 (35.0%, including 12 patients who died) patients experienced disease recurrence. Of these 63 patients, 42.9% (27 of 63, including 12 patients who died) had progression, and 30.1% (19 of 63, including 12 patients who died) developed metastatic disease. It was found that the patients with IMP3 expression had lower disease-free (*P* < .001), progression-free (*P* = .002) and metastasis-free (*P* = .019) survival rates compared with those without IMP3 expression (Fig. 2). However, we did not find apparent differences in the overall survival rate (*P* = .058) between the IMP3-positive and IMP3-negative groups.

**Figure 3:**
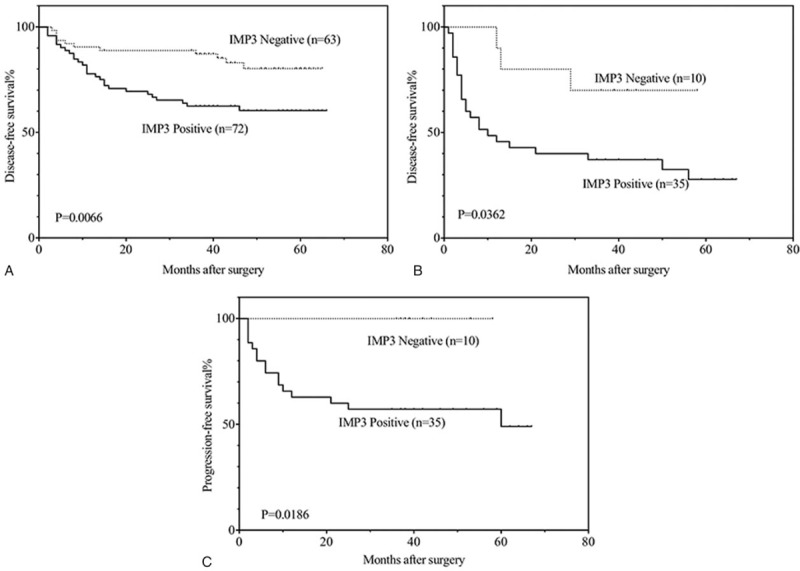
Kaplan–Meier analysis of disease-free survival (A) in patients with Ta disease and disease-free (B), progression-free (C) survival in patients with T1 disease.

**Figure 4:**
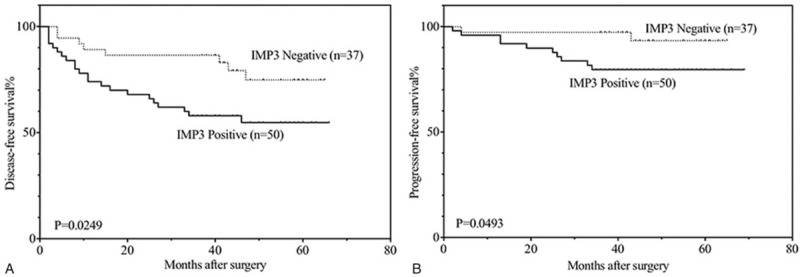
Kaplan–Meier analysis of disease-free (A), progression-free (B) survival in patients with low grade disease.

**Figure 5:**
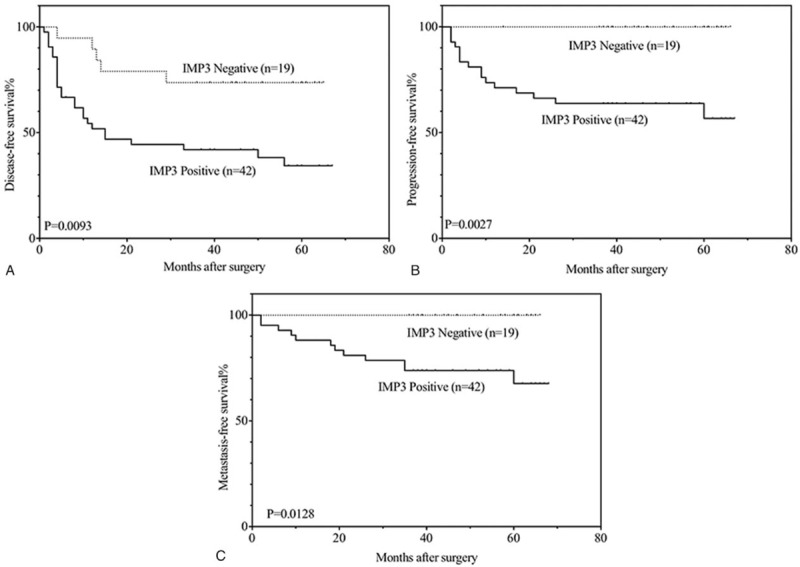
Kaplan–Meier analysis of disease-free (A), progression-free (B), disease-free (C) survival in patients with high grade disease.

Figure 3 indicates that IMP3-positive patients had an obviously unfavorable disease- free (3a: *P* = .007) survival compared with IMP3-negative members with Ta disease. Additionally, IMP3-positive patients with stage T1 tumors had a significantly unfavorable disease-free (3b: *P* = .036) and progression-free (3c: *P* = .019) survival relative to those who were IMP3-negative. In patients with low-grade non-muscle- invasive urothelial carcinomas, IMP3 expression also proved to be an unfavorable prognostic factor of tumor recurrence (Fig. 4a: *P* = .025) and progression (Fig. 4b: *P* = .049). The same results in recurrence (Fig. 5a: *P* = .009), progression (Fig. 5b: *P* = .003) and metastasis (Fig. 5c: *P* = .013) were also found in patients with high grade disease.

### 3.4 Multivariate analysis

Table 2 shows the result of multivariable Cox regression analysis in 180 patients with non-muscle-invasive urothelial carcinomas of the bladder. In this analysis, adjusting for age, gender, tumor size, tumor multiplicity, tumor stage and grade, and smoking history, the IMP3 status in patients with non-muscle-invasive urothelial carcinomas was still independently related to the disease-free survival (hazard ratio (HR): 2.52; 95% confidence interval (CI), 1.39–4.56; *P* = .002), progression-free survival (HR: 5.19; 95% CI, 1.54–17.46; *P* = .008) and metastasis-free survival (HR:4.87; 95% CI, 1.08– 22.02; *P* = .040) rates. In addition to the expression of IMP3, tumor multiplicity is also a risk factor affecting disease-free survival, and tumor stage T1 (relative to stage Ta) is an unfavorable prognostic factor of progression-free survival and metastasis-free survival.

**Table 2:**
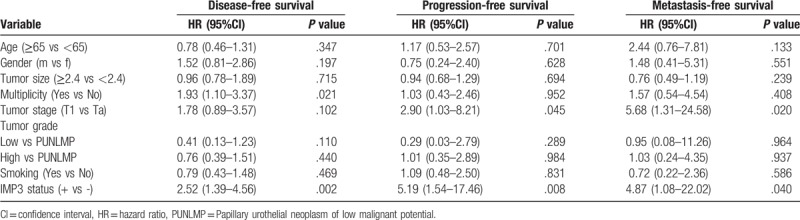
Multivariable analysis for disease-free, progression-free and metastasis-free survivals of non-muscle-invasive urothelial carcinomas.

## 4 Discussion

Bladder cancer is still a major epidemiologic problem, and the incidence continues to rise each year. Patients with non-muscle-invasive urothelial carcinoma represent a clinically heterogeneous group with varying risk of disease relapse. The high rate of recurrence is the feature of bladder cancer that makes follow-up a crucial component in effective management. After transurethral resection of the tumor, patients should have regular cystoscopy and intravesical chemotherapy in case of recurrence and progression. Several studies have identified pathological risk factors for recurrence and progression of non-muscle-invasive urothelial carcinoma of the bladder, including tumor grade, stage, multiplicity, and size.^[18,19]^ However, despite the benefits in prevention of recurrence, a similar benefit for the prevention of progression was not seen.^[20]^

IMP3 appears to be a growth-related gene and is highly expressed in a variety of tumors. In most studies of tumors so far, the expression of IMP3 is congruously related to the advanced stage and aggressive behavior of tumors. In our study, we determined the expression of IMP3 in 203 urothelial carcinomas (183 non-muscle-invasive and 20 muscle-invasive) and 20 benign tissues adjacent to cancer tissue by IHC. We found that the expression of IMP3 is significantly higher in muscle-invasive urothelial carcinomas than in non-muscle-invasive urothelial carcinomas, and among those with non-muscle- invasive urothelial carcinoma, IMP3 is more often expressed in high stage and grade urothelial tumors but less in low stage and grade urothelial tumors and not in benign urothelial specimens. Combined with the in vitro studies demonstrating a crucial role of IMP3 in the tumor proliferation and invasion,^[15,16]^ our findings further confirm that the overexpression of this protein is not involved in tumor initiation but may be involved in tumor progression.

As we expected, our findings indicated that tissue expression of IMP3 was highly related to advanced tumor grade and stage and to tumor recurrence in non-muscle-invasive urothelial carcinoma. It is acknowledged that these clinical indicators are directly associated with tumor prognosis. Furthermore, IMP3-positive patients presented a noteworthy unfavorable prognostic factor of progression relative to those with IMP3-negative diseases. The current study showed 22.4% (24 of 107) of patients with IMP3-positive tumors developed a higher stage in relation to only 4.1% (3 of 73) of patients with IMP3-negative tumors. In the multivariable analysis, IMP3-positive patients with non-muscle-invasive urothelial carcinomas were 5.2 times more likely than patients with IMP3-negative carcinomas to develop subsequent invasive lesions, even after adjusting for other proverbial clinical variables such as tumor stage and grade. Consequently, our results strongly showed that IMP3-positive patients with non- muscle-invasive urothelial carcinomas should receive aggressive treatment and positive follow-up compared with IMP3-negative patients.

We also recognized that IMP3 expression was highly independently associated with disease metastasis in non-muscle-invasive urothelial carcinomas. Distant metastasis is always considered as the primary reason for death and therapeutic failure in bladder urothelial carcinoma patients. Our data showed that 15.0% (16 of 107) of patients with IMP3-positive tumors developed metastasis, in contrast with 4.1% (3 of 73) of patients with IMP3-negative ones. Patients with IMP3 expression had an overall 4.9-times increased risk of disease metastasis after adjusting for other well-known clinicopathological features. We also realized that during the IMP3-positive patients who developed metastasis, 70.6% had stage T1 tumors. There is a view that patients with advanced grade T1 tumors should receive radical cystectomy.^[21,22]^ Thus, it is a difficult clinical decision for high-risk patients with stage T1 tumors to choose transurethral resection of the tumor with postoperative intravesical chemotherapy or radical cystectomy. Our results suggest that positive IMP3 expression was significantly related to poor survival, and these patients can consider direct radical cystectomy, while patients without IMP3 expression could be considered for transurethral resection of the tumor with postoperative intravesical chemotherapy.

However, several limitations should be taken into consideration. From the current study, IMP3 can be said to be related to poor prognosis in non-muscle-invasive urothelial carcinoma, but the mechanism of how IMP3 influences the biological behavior of non-muscle-invasive urothelial carcinomas is unclear. Before it can be used as a target for molecular diagnosis and treatment, more research is needed. This study design was retrospective, which has inherent biases and confounders, and a limited follow-up period. Also, to our regret, there were no significant differences in the overall survival rates. We know that patients with non-muscle-invasive urothelial carcinomas of the bladder have a relatively long survival period, which could be a potential reason for this. We will continue to pay more attention to these patients. In addition, this study did not analyze muscle-invasive bladder cancer patients, so we will increase the sample size for further study of invasive bladder cancer.

## 5 Conclusions

In general, IMP3 is a promising prognostic biomarker for non-muscle-invasive urothelial carcinoma. IMP3 status may bring critical prognostic information for patients with non-muscle-invasive urothelial carcinomas and will give huge assistance to physicians in identifying patients with an unfavorable prognosis who might benefit from early, aggressive and individualized therapy after transurethral resection. Further studies are required to elucidate the molecular mechanisms and pathways through which IMP3 affects the biological phenotype of non-muscle-invasive urothelial carcinoma.

## Author contributions

FY, QZ and NX designed the study and edit the manuscript. FY, QZ and LM carried out data acquisition and analysis. FY, QZ and LM performed the majority of the experiments. FY and QZ wrote the manuscript. FY and QZ collected the clinical samples and managed the clinical data. All authors read and approved the final manuscript.

**Conceptualization:** Feiya Yang, Qiang Zhou, Lingquan Meng, Nianzeng Xing.

**Data curation:** Feiya Yang, Qiang Zhou, Lingquan Meng, Nianzeng Xing.

**Formal analysis:** Feiya Yang, Qiang Zhou, Lingquan Meng, Nianzeng Xing.

**Funding acquisition:** Nianzeng Xing.

**Investigation:** Feiya Yang, Qiang Zhou, Nianzeng Xing.

**Methodology:** Feiya Yang, Qiang Zhou, Nianzeng Xing.

**Project administration:** Feiya Yang, Qiang Zhou, Nianzeng Xing.

**Resources:** Feiya Yang, Qiang Zhou, Lingquan Meng, Nianzeng Xing.

**Software:** Feiya Yang, Qiang Zhou, Nianzeng Xing.

**Supervision:** Nianzeng Xing.

**Validation:** Feiya Yang, Qiang Zhou, Nianzeng Xing.

**Visualization:** Feiya Yang, Qiang Zhou, Nianzeng Xing.

**Writing – original draft:** Feiya Yang, Qiang Zhou, Nianzeng Xing.

**Writing – review & editing:** Feiya Yang, Qiang Zhou, Nianzeng Xing.
